# Effect of endometrial thickness changes on clinical pregnancy rates after progesterone administration in a single frozen-thawed euploid blastocyst transfer cycle using natural cycles with luteal support for PGT-SR- and PGT-M-assisted reproduction: a retrospective cohort study

**DOI:** 10.1186/s12958-021-00841-x

**Published:** 2021-10-09

**Authors:** Ziqi Jin, Jingdi Li, EnTong Yang, Hao Shi, Zhiqin Bu, Wenbin Niu, Fang Wang, Mingzhu Huo, Hui Song, YiLe Zhang

**Affiliations:** 1 Reproductive Medicine Center, First Affiliated Hospital of Zhengzhou University, Zhengzhou, China; 2 Zhengzhou, People’s Republic of China

**Keywords:** Endometrial thickness change ratio, Euploid blastocyst transfer, Clinical pregnancy rates, Natural cycle, Transvaginal ultrasound

## Abstract

**Background:**

To investigate whether the endometrial thickness change ratio from the progesterone administration day to the blastocyst transfer day is associated with pregnancy outcomes in a single frozen-thawed euploid blastocyst transfer cycle.

**Methods:**

All patients used natural cycles with luteal support for endometrial preparation and selected a single euploid blastocyst for transfer after a biopsy for preimplantation genetic testing. The endometrial thickness was measured by transvaginal ultrasound on the progesterone administration day and the transfer day, the change in endometrial thickness was measured, and the endometrial thickness change ratio was calculated. According to the change rate of endometrial thickness, the patients were divided into three groups: the endometrial thickness compaction group, endometrial thickness non-change group and endometrial thickness expansion group. Among them, the endometrial thickness non-change and expansion groups were combined into the endometrial thickness noncompaction group.

**Results:**

Ultrasound images of the endometrium in 219 frozen-thawed euploid blastocyst transfer cycles were evaluated. The clinical pregnancy rate increased with the increase in endometrial thickness change ratio, while the miscarriage rate and live birth rate were comparable among the groups. The multiple logistic regression results showed that in the fully adjusted model a higher endometrial thickness change ratio (per 10%) was associated with a higher clinical pregnancy rate (adjusted odds ratio [aOR] 1.29; 95% confidence interval [CI], 1.01–1.64; *P* = .040). Similarly, when the patients were divided into three groups according to the change rate of endometrial thickness, the endometrial thickness noncompaction group had a significant positive effect on the clinical pregnancy rate compared with the endometrial thickness compaction group after adjusting for all covariates.

**Conclusions:**

In frozen-thawed euploid blastocyst transfer cycles in which the endometrium was prepared by natural cycles with luteal support, the clinical pregnancy rate was higher in cycles without endometrial compaction after progesterone administration.

**Supplementary Information:**

The online version contains supplementary material available at 10.1186/s12958-021-00841-x.

## Introduction

Embryo implantation is a complicated process, and the key factors affecting embryo implantation are embryo quality and endometrial receptivity [1]. In recent years, with the development of preimplantation genetic testing (PGT) technology, selecting euploid embryos for transfer by biopsy based on cytogenetics and/or molecular biology techniques before implantation has become possible [2]. Despite the optimization of embryo quality, endometrial receptivity disorders are still a prominent problem that affect pregnancy success and result in approximately two-thirds of implantation failures [3, 4]. When the embryo is implanted into the nonreceptive endometrium, it will lead to implantation failure. Therefore, identifying the “window of implantation” (WOI) of the endometrium by various techniques is very important.

At present, transvaginal ultrasound has become a routine examination method for evaluating the endometrium because of its convenience and noninvasive characteristics. Endometrial receptivity is usually identified by several sonographic parameters, such as endometrial thickness [5–7], endometrial volume [8], endometrial pattern [9, 10] and endometrial blood flow [11, 12], among which endometrial thickness has been widely accepted as an indicator to predict endometrial receptivity [13]. However, whether endometrial thickness can predict pregnancy outcomes is still controversial [6, 14–16].

In previous studies, the monitoring of endometrial thickness was mostly concentrated around the human chorionic gonadotrophin (hCG) trigger day in fresh cycles [6, 16, 17] or at the end of the endometrium proliferation phase in the frozen-thawed embryo transfer (FET) cycles [15–17], while research on endometrial thickness in the luteal phase around the embryo transfer day was relatively rare [15, 18, 19]. However, the embryo transfer day is usually considered to be within the WOI, so assuming that the endometrial condition on the day of transfer is more representative of endometrial receptivity is reasonable. In addition, the pattern and structure of the endometrium change throughout the menstrual cycle [15], but few studies have investigated the change in endometrial thickness after progesterone administration, and the conclusions are contradictory [20–24]. To our knowledge, no related research is available on euploid blastocyst transfer to the endometrium prepared by a natural cycle.

The purpose of this study was to investigate the effect of endometrial thickness changes measured by transvaginal ultrasound after progesterone administration on clinical pregnancy rates in women with their first single frozen-thawed euploid blastocyst transfer cycles. Considering the differences between different endometrial preparation protocols, we only chose natural cycles with luteal support and without exogenous estrogen for endometrial preparation to reduce the factors that may affect the endometrium in each cycle and ensure the consistency of the study.

## Methods

### Study design and population

As patients with chromosome translocation or monogenic diseases are the main sources of preimplantation genetic diagnosis (PGD) treatment in our center, this retrospective cohort study included women who received PGT for chromosomal structural rearrangements (PGT-SR) or PGT for monogenic/single gene defect (PGT-M) treatment and used natural cycles with luteal support to prepare the endometrium for the first single frozen-thawed euploid blastocyst transfer in the Reproductive Medical Center of the First Affiliated Hospital of Zhengzhou University from January 2014 to December 2019. The indications for PGT-SR and PGT-M included in this article are listed in Supplementary Table 1. The study data were extracted from the Clinical Reproductive Medicine Management System/Electronic Medical Record Cohort Database (CCRM/EMRCD). All patients were followed up for at least 1 year. This study was approved by the Institutional Review Board and Ethics Committee of the First Affiliated Hospital of Zhengzhou University. Because of the retrospective character of the study, informed consent was waived.

Briefly, this study recruited 233 couples where the women had chromosome translocation or monogenic disease while the men were normal. They received PGD treatment and underwent the first single frozen-thawed euploid blastocyst transfer from January 2014 to December 2019. Patients who met one of the following criteria were excluded: (1) routine hysteroscopy before the beginning of PGD cycles showed the presence of uterine pathology, (2) endometriosis, (3) a lower endometrial thickness (< 7 mm) on the day of progesterone administration, or (4) missing endometrial data. The resulting cohort obtained in this study included 219 participants for the final analysis.

### Treatment protocol

All patients were treated with controlled ovarian stimulation with a short-acting gonadotropin-releasing hormone agonist long protocol (MLSL) in the mid-luteal phase, and the mature oocytes were fertilized by intracytoplasmic sperm injection (ICSI) after oocyte retrieval. The specific details of the protocol and the operation method of ICSI have been described previously [25]. On the morning of the fifth or sixth day after oocyte retrieval, experienced embryologists scored the blastocysts according to the system of Gardner and Schoolcraft under consistent laboratory conditions. Embryologists selected blastocysts of grade 3 BC and above, and 3–5 trophectoderm (TE) cells were biopsied by laser irradiation. According to the standard protocol provided by the QIAGEN REPLI-g Single Cell kit, whole-genome amplification of TE cells obtained by biopsy was conducted, and the specific steps were as described previously [26]. The biopsied blastocysts were vitrified and preserved in liquid nitrogen.

All endometrial preparation protocols of frozen-thawed blastocyst transfer used natural cycles with luteal support and without exogenous estrogen. Transvaginal ultrasound was performed on days 8 to 9 of the menstrual cycle to monitor follicular growth. When the dominant follicle reached a mean diameter of 14 ~ 16 mm, blood samples were taken every day to monitor hormone levels. The date of ovulation was determined by monitoring the levels of serum estradiol, progesterone and luteinizing hormone, combined with transvaginal ultrasound to identify follicular rupture. Intramuscular injection of progesterone (40 mg) was administered from the day of ovulation, and oral dydrogesterone (10 mg) ([this dose changed to 20 mg after 2 days] [duphaston]; Solvay Pharmaceuticals B.V., Veenendaal, the Netherlands) was started 1 day after ovulation. Blastocyst transfer was performed 5 days after ovulation. From the date of transfer, 90 mg vaginal progesterone gel (Xenotong, Merck Sherano, Switzerland) and 20 mg oral dydrogesterone were administered daily. Two weeks after blastocyst transfer, serum hCG was detected to evaluate the outcome of FET. Transvaginal ultrasound was performed 5 weeks after blastocyst transfer to confirm clinical pregnancy.

### Endometrial thickness assessment

The ovulation date was determined by transvaginal ultrasonography, and endometrial thickness was recorded. On the morning of the blastocyst transfer day, the endometrium was reevaluated by transvaginal ultrasound, and the thickness was recorded. Patients with normal endometrium on ultrasound imaging were arranged for blastocyst transfer. All ultrasound examinations were performed by a group of uniformly trained and experienced ultrasound technicians using the same ultrasound equipment.

According to the difference in endometrial thickness on the day of blastocyst transfer and progesterone administration, the patients were divided into three groups: the endometrial lining compaction group (compaction group), endometrial lining expansion group (expansion group) and non-change endometrial group (non-change group). The endometrial thickness change ratio was defined as the difference in endometrial thickness on the transfer day and the progesterone administration day divided by the endometrial thickness on the progesterone administration day.

### Definition of clinical outcomes

We used the consensus reached by the American Society for Reproductive Medicine in 2017 to define clinical outcomes [27]. Clinical pregnancy was defined as the detection of one or more gestational sacs by ultrasound. Miscarriage was defined as spontaneous abortion of an intrauterine pregnancy before 22 weeks of pregnancy. Live birth was defined as the delivery of at least one live-birth baby after 22 weeks of pregnancy.

### Statistical analysis

The baseline characteristics of the patients were described. For continuous variables, the data are expressed as the mean ± standard deviation (normal distribution) or median (interquartile range [IQR]) (skewed distribution); for categorical variables, the data are expressed as the frequency or percentage. The significant difference in the endometrial thickness change rate among the three groups was tested by one-way ANOVA for continuous variables and chi-squared tests for categorical variables.

Univariate analysis was used to evaluate the influence of each variable on the clinical pregnancy rate. We used a multivariate logistic regression model to calculate the crude odds ratios (ORs) and adjusted odds ratios (aORs) of 95% confidence intervals (CIs) and analyzed the relationship between the endometrial thickness change ratio and the pregnancy outcome. Both the unadjusted model and the multivariable adjustment model were used. If covariates changed the estimated value of the endometrial thickness change ratio on the clinical pregnancy rate by more than 10%, were significantly related to the clinical pregnancy rate, or were based on recently published studies and clinical experience, covariates were included in the final model as potential confounders. In this study, multivariate logistic regression models were adjusted for female age at blastocyst transfer, body mass index (BMI), infertility duration, infertility type, anti-Müllerian hormone (AMH), genetic category and estradiol on the day of progesterone administration. The trend test used linear regression to input the median value of each endometrial thickness change group (compaction group, non-change group and expansion group) as a continuous variable into the models.

To avoid reduced statistical power and bias caused by the direct exclusion of missing data, we used multiple imputation, based on 5 replications and the Markov chain Monte Carlo method in the R MI procedure [28], to account for missing data on AMH and estradiol on the day of progesterone administration. Adjustment for confounders in the multiple regression analysis was performed on the imputed data to compare the results with the results obtained by using the full data cohort.

**Fig. 1:**
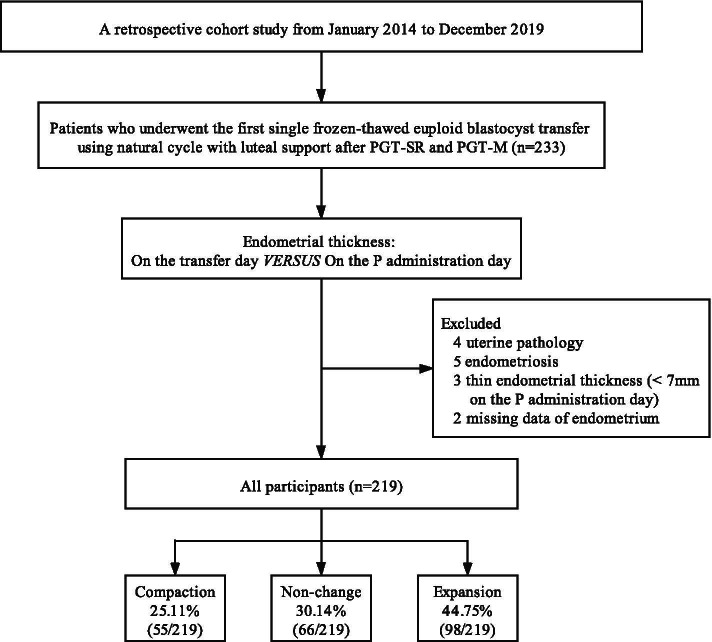
Flowchart of patients. *Note:* PGT-SR = preimplantation genetic testing for chromosomal structural rearrangements; PGT-M = preimplantation genetic testing for monogenic/single gene defects; P = progesterone

The data collection method of this study is shown in Fig. 1. All statistical analyses were performed with the statistical software package R version 3.4.3 (The R Foundation, Vienna, Austria). A two-sided significance level of 0.05 was used to evaluate the statistical significance.

## Results

### Characteristics of the study cohort

In this retrospective cohort study, a total of 233 patients received their first single frozen-thawed euploid blastocyst transfer, which used natural cycles to prepare the endometrium. Among the cases, 4 had uterine pathology, 5 had endometriosis, 3 had a very thin endometrium (< 7 mm on the progesterone administration day), and 2 had missing endometrial data. Finally, this study included 219 eligible patients with a natural cycle (Fig. 1). In all frozen-thawed euploid blastocyst transfer cycles, the endometrial thickness decreased in 25.11% of patients, increased in 44.75% of patients, and had no change in the remaining 30.14% of patients on the day of blastocyst transfer.

### Comparison of differences between groups

The baseline characteristics of all participants are shown in Table 1. The expansion group was younger and had a lower BMI. However, in terms of infertility duration, infertility types, gravidity, parity, basal serum follicle-stimulating hormone, AMH, hormone level on progestogen administration day, blastocyst grade, day of embryo development at transfer, miscarriage rate and live birth rate, the three groups were comparable.

**Table 1:** Baseline characteristics and pregnancy outcomes of patients with single frozen-thawed euploid blastocyst transfer

Characteristics	Total	Compaction	Non-change	Expansion	***P*** value
N	219	55 (25.11%)	66 (30.14%)	98 (44.75%)	
Female age at oocyte retrieval (y)	30.21 ± 3.87	30.85 ± 4.40	30.88 ± 3.85	29.41 ± 3.44	0.021
BMI (kg/m2)	22.47 ± 2.66	22.97 ± 2.59	22.84 ± 2.59	21.93 ± 2.67	0.025
Infertility duration (y)	2.00 (1.00–4.00)	2.00 (1.00–4.00)	2.00 (1.00–4.00)	2.00 (1.00–3.80)	0.630
Infertility type (%)					0.943
Primary	60 (27.40%)	16 (29.09%)	18 (27.27%)	26 (26.53%)	
Secondary	159 (72.60%)	39 (70.91%)	48 (72.73%)	72 (73.47%)	
Gravidity	2.00 (0.00, 3.00)	2.00 (0.00, 3.00)	2.00 (0.00, 2.00)	2.00 (0.00, 2.00)	0.277
Parity, Mean ± SD (Range)	0.48 ± 0.71 (0.00–3.00)	0.55 ± 0.77 (0.00–3.00)	0.48 ± 0.73 (0.00–3.00)	0.45 ± 0.66 (0.00–3.00)	0.722
No. of miscarriages	1.00 (0.00, 2.00)	1.00 (0.00, 2.00)	1.00 (0.00, 2.00)	1.00 (0.00, 2.00)	0.418
Basal serum FSH (mIU/ml)	6.80 ± 1.99	6.95 ± 2.82	6.98 ± 1.78	6.58 ± 1.49	0.368
AMH (ng/ml)	3.00 (1.90, 4.50)	3.30 (2.10, 5.20)	2.70 (1.90, 3.90)	3.00 (2.10, 5.00)	0.082
No. of retrieved oocytes	16.00 (12.00, 21.00)	16.00 (12.00, 21.00)	15.50 (12.20, 20.00)	17.00 (13.00, 21.80)	0.686
Genetic category (%)					0.548
Reciprocal translocation	118 (53.88%)	33 (60.00%)	35 (53.03%)	50 (51.02%)	
Robertsonian translocation	41 (18.72%)	6 (10.91%)	14 (21.21%)	21 (21.43%)	
Single gene disorders	60 (27.40%)	16 (29.09%)	17 (25.76%)	27 (27.55%)	
Female age at blastocyst transfer (y)	30.49 ± 3.80	31.13 ± 4.33	31.12 ± 3.76	29.71 ± 3.39	0.024
Endometrial thickness on the day of P administration (mm)	10.18 ± 1.99	11.16 ± 2.00	10.20 ± 2.09	9.62 ± 1.71	< 0.001
Endometrial thickness at transfer (mm)	10.45 ± 2.01	9.60 ± 1.68	10.20 ± 2.09	11.09 ± 1.92	< 0.001
Triple-line endometrial pattern on the day of P administration (%)					0.856
A	196 (89.50%)	50 (90.91%)	58 (87.88%)	88 (89.80%)	
B	23 (10.50%)	5 (9.09%)	8 (12.12%)	10 (10.20%)	
E2 on the day of P administration (pg/mL)	124.20 (83.50, 174.80)	127.40 (92.40, 212.00)	122.80 (82.20, 155.50)	117.70 (80.40, 171.60)	0.226
LH on the day of P administration (mIU/ml)	19.30 (12.70, 29.20)	20.70 (14.40, 31.10)	19.70 (12.20, 31.40)	18.10 (12.10, 26.90)	0.744
P on the day of P administration (ng/ml)	1.10 (0.90, 1.40)	1.10 (0.80, 1.30)	1.10 (0.90, 1.40)	1.20 (1.00, 1.60)	0.094
Embryo expansion grade at transfer	3.42 ± 0.86	3.46 ± 0.95	3.52 ± 0.95	3.33 ± 0.74	0.353
Embryo inner cell mass grade at transfer (%)					0.236
A	15 (6.88%)	1 (1.85%)	6 (9.09%)	8 (8.16%)	
B	203 (93.12%)	53 (98.15%)	60 (90.91%)	90 (91.84%)	
Embryo trophectoderm grade at transfer (%)					0.699
A	1 (0.46%)	0 (0.00%)	0 (0.00%)	1 (1.02%)	
B	117 (53.67%)	26 (48.15%)	37 (56.06%)	54 (55.10%)	
C	100 (45.87%)	28 (51.85%)	29 (43.94%)	43 (43.88%)	
Morphology score (%)					0.281
< 4 BC	147 (67.43%)	36 (66.67%)	40 (60.61%)	71 (72.45%)	
≥ 4 BC	71 (32.57%)	18 (33.33%)	26 (39.39%)	27 (27.55%)	
Day of embryo development at transfer (%)					0.706
Day 5	156 (71.56%)	38 (69.09%)	49 (75.38%)	69 (70.41%)	
Day 6	62 (28.44%)	17 (30.91%)	16 (24.62%)	29 (29.59%)	
Clinical pregnancy rate (%)	131 (59.82%)	25 (45.45%)	42 (63.64%)	64 (65.31%)	0.042
Miscarriage rate (%)	21 (16.03%)	2 (8.00%)	5 (11.90%)	14 (21.88%)	0.187
Live-birth rate (%)	111 (56.63%)	23 (46.94%)	37 (64.91%)	51 (56.67%)	0.177

*Note: BMI* body mass index, *FSH* follicle-stimulating hormone, *AMH* anti-Müllerian hormone, *P* progesterone, *E2* estradiol, *LH* luteinizing hormone

**Fig. 2:**
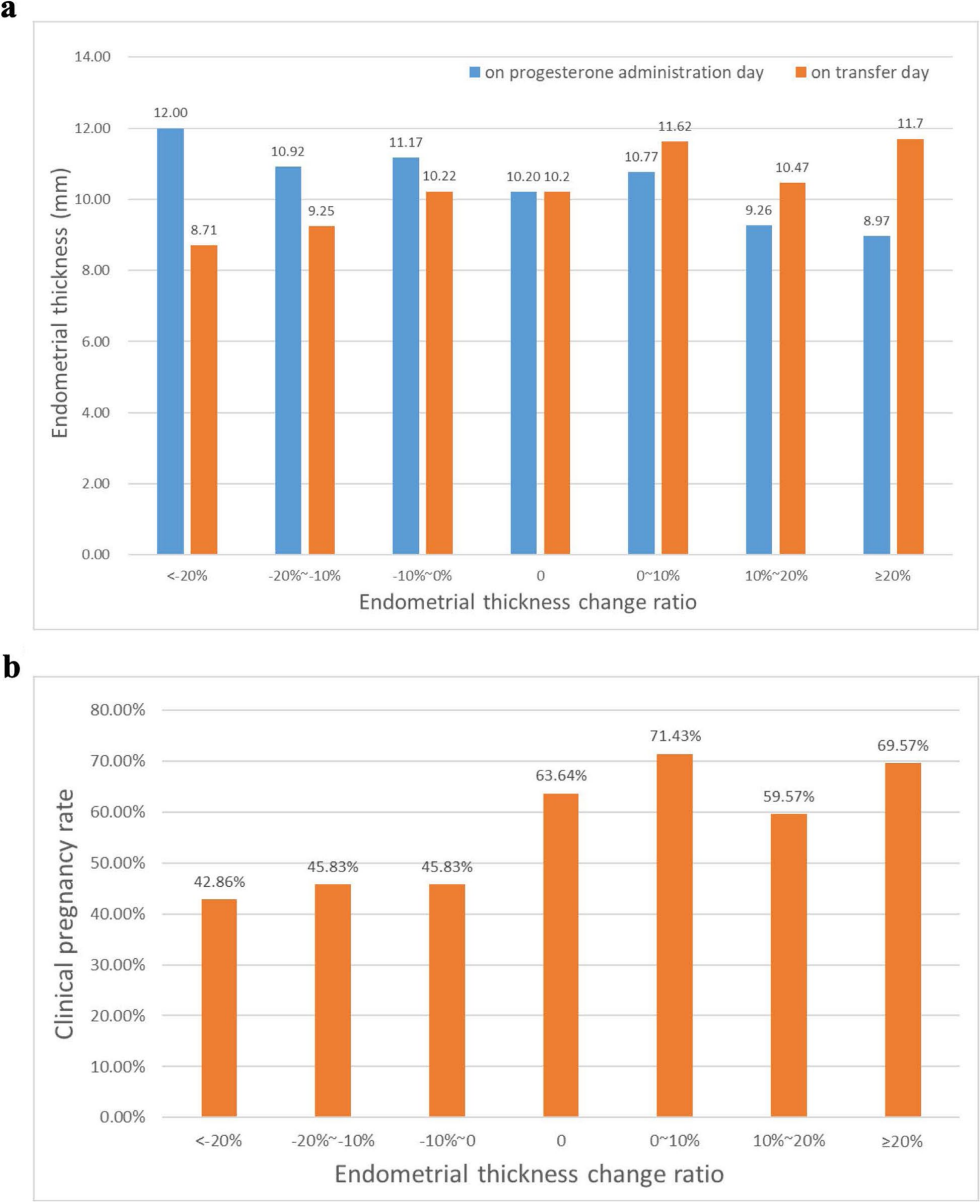
**a** Endometrial thickness on the progesterone administration day and blastocyst transfer day in different endometrial thickness change ratio groups. **b** Relationship between endometrial thickness change ratio and clinical pregnancy rate

In all participants, the average endometrial thickness on the progesterone administration day was 10.18 ± 1.99 mm, and on the transfer day it was 10.45 ± 2.01 mm, showing a trend of increased endometrial thickness after progesterone administration. Compared with the other two groups, the endometrial thickness in the expansion group was the thinnest on the day of progesterone administration (11.16 ± 2.00 vs. 10.20 ± 2.09 vs. 9.62 ± 1.71 mm, *P* < .001) and then increased sharply, becoming the thickest group on the day of blastocyst transfer (9.60 ± 1.68 vs. 10.20 ± 2.09 vs. 11.09 ± 1.92, *P* < .001). In contrast, endometrial thickness in the compaction group ranged from the thickest on the day of progesterone administration to the thinnest on the day of transfer. The patients were subdivided according to the endometrial thickness change ratio, showing similar results **(**Fig. 2a**)**.

The average clinical pregnancy rate of patients was 59.82%, among which the clinical pregnancy rate of the compaction group was significantly lower than that of the non-change group and the expansion group (45.45% vs. 63.64% vs. 65.31%, *P* = .042). The patients were also subdivided according to the endometrial thickness change ratio. The clinical pregnancy rate was only 42.86% in the subgroup where endometrial thickness decreased by ≥20%, while it was nearly 70% in the subgroup where endometrial thickness increased by ≥20% group (Fig. 2b).

### Association of endometrial thickness change ratio with clinical pregnancy rates

A univariate regression analysis was used to evaluate the influence of each variable on the clinical pregnancy rates (Table 2). Overall, the endometrial thickness change ratio was positively associated with the clinical pregnancy rate.

**Table 2:** Univariate analysis for clinical pregnancy rate

Covariate	OR (95% CI)	***P*** value
Female age at oocyte retrieval (y)	0.98 (0.91, 1.05)	0.590
BMI (kg/m2)	0.98 (0.89, 1.09)	0.707
Infertility duration (y)	0.94 (0.85, 1.05)	0.281
Infertility type		
Primary	Reference	
Secondary	0.90 (0.49, 1.65)	0.732
Gravidity	0.94 (0.79, 1.13)	0.532
Parity	0.85 (0.58, 1.24)	0.390
No. of miscarriages	0.99 (0.80, 1.22)	0.902
Basal serum FSH (mIU/ml)	0.93 (0.81, 1.07)	0.322
AMH (ng/ml)	1.07 (0.94, 1.22)	0.290
No. of retrieved oocytes	1.00 (0.97, 1.04)	0.951
Genetic category		
Reciprocal translocation	Reference	
Robertsonian translocation	0.85 (0.41, 1.74)	0.648
Single gene disorders	1.06 (0.56, 2.01)	0.847
Female age at blastocyst transfer (y)	0.98 (0.91, 1.05)	0.596
Endometrial thickness on the day of P administration (mm)	0.93 (0.81, 1.07)	0.295
Endometrial thickness at transfer (mm)	1.04 (0.91, 1.19)	0.556
Triple-line endometrial pattern on the day of P administration		
A	Reference	
B	0.86 (0.36, 2.06)	0.734
Endometrial thickness change ratio after P administration	1.02 (1.00, 1.04)	0.034
E2 on the day of P administration (pg/mL)	1.00 (1.00, 1.00)	0.492
LH on the day of P administration (mIU/ml)	1.00 (0.98, 1.02)	0.970
P on the day of P administration (ng/ml)	0.99 (0.55, 1.79)	0.972
Morphology score		
< 4 BC	Reference	
≥ 4 BC	1.23 (0.68, 2.20)	0.491
Day of embryo development at transfer		
Day 5	Reference	
Day 6	0.76 (0.42, 1.38)	0.364

*Note: BMI* body mass index, *FSH* follicle-stimulating hormone, *AMH* anti-Müllerian hormone, *P* progesterone, *E2* estradiol, *LH* luteinizing hormone, *OR* odds ratio, *CI* confidence interval

Multivariate logistic regression models were used to evaluate whether the endometrial thickness change ratio had an independent effect on the clinical pregnancy rate (Table 3). When the endometrial thickness change ratio was taken as a continuous variable, a higher endometrial thickness change ratio (per 10%) was associated with a higher clinical pregnancy rate, whether in the unadjusted model (OR 1.24; 95% CI, 1.02–1.52; *P* = .034) or in the model with the adjustment for female age at blastocyst transfer, BMI, infertility duration, infertility type, AMH, genetic category and estradiol on the day of progesterone administration (aOR 1.29; 95% CI, 1.01–1.64; *P* = .040). According to the change in endometrial thickness, the patients were divided into three groups as categorical variables, namely, the compaction, non-change and expansion groups, and the non-change group was used as the reference. In the unadjusted model, the clinical pregnancy rate of the compaction group was significantly lower than that of the non-change group (OR 0.48; 95% CI, 0.23–0.99; *P* = .046), and that of the expansion group was 8% higher than that of the reference group, but the difference was not significant (OR 1.08; 95% CI, 0.56–2.06; *P* = .826) (*P* for trend = 0.025). Similarly, after adjusting for all covariates and taking the non-change group as the reference group, the clinical pregnancy rate in the compaction group was 59% lower than that of the reference group (aOR 0.41; 95% CI, 0.17–0.99; *P* = .049); meanwhile, no significant difference was found between the expansion group and the reference group (aOR 1.13; 95% CI, 0.54–2.37; *P* = .753) (*P* for trend = 0.025). Combining the non-change group and the expansion group into one group (data not shown), the results showed that compared with the endometrial thickness compaction, the clinical pregnancy rate of the endometrial thickness noncompaction (including the non-change and expansion groups) increased by 1.55 times (OR 2.55; 95% CI, 1.17–5.58; *P* = .019).

**Table 3:** Association between endometrial thickness change ratio and clinical pregnancy rate in different models

	Crude model^a^		Adjusted model I^**b**^		Adjusted model II^**c**^	
	OR (95% CI)	***P*** value	OR (95% CI)	***P*** value	OR (95% CI)	***P*** value
Endometrial thickness change ratio (per 10%)	1.24 (1.02, 1.52)	0.034	1.25 (1.01, 1.54)	0.036	1.29 (1.01, 1.64)	0.040
Grouping of endometrial thickness						
= 0 (non-change)	Reference		Reference		Reference	
< 0 (compaction)	0.48 (0.23,0.99)	0.046	0.47 (0.22,0.97)	0.042	0.41 (0.17,0.99)	0.049
> 0 (expansion)	1.08 (0.56,2.06)	0.826	1.05 (0.54,2.06)	0.876	1.13 (0.54,2.37)	0.753
P for trend	0.025		0.028		0.025	

*Note: BMI* body mass index, *AMH* anti-Müllerian hormone, *E2* estradiol, *P* progesterone, *OR* odds ratio, *CI* confidence interval

^a^No adjustments for other covariates

^b^Adjusted for female age at transfer, BMI, infertility duration and infertility type

^c^Adjusted for all covariables in model I plus AMH, E2 on the day of P administration and genetic category

After addressing the missing data with multiple imputation, the results of the multivariate logistic regression analyses with multiple adjustment strategies were similar to those of participants with complete data (Table 4).

**Table 4:** Association between endometrial thickness change ratio and clinical pregnancy rate in different models — by using data after multiple imputation

	Crude model^**a**^		Adjusted model I^**b**^		Adjusted model II^**c**^	
	OR (95% CI)	***P*** value	OR (95% CI)	***P*** value	OR (95% CI)	***P*** value
Endometrial thickness change ratio (per 10%)	1.24 (1.02, 1.52)	0.034	1.24 (1.01, 1.53)	0.041	1.25 (1.02, 1.54)	0.036
Grouping of endometrial thickness						
= 0 (non-change)	Reference		Reference		Reference	
< 0 (compaction)	0.48 (0.23,0.99)	0.046	0.47 (0.23,0.99)	0.047	0.44 (0.21,0.93)	0.032
> 0 (expansion)	1.08 (0.56,2.06)	0.826	1.04 (0.54,2.02)	0.899	1.03 (0.53,2.01)	0.935
P for trend	0.025		0.028		0.018	

*Note BMI* body mass index, *AMH* anti-Müllerian hormone, *E2* estradiol, *P* progesterone, *OR* odds ratio, *CI* confidence interval.

^a^No adjustments for other covariates

^b^Adjusted for female age at transfer, BMI, infertility duration and infertility type

^c^Adjusted for all covariables in model I plus AMH, E2 on the day of P administration and genetic category

## Discussion

To date, although many studies on endometrial thickness have been conducted, few studies have evaluated the effect of endometrial thickness changes on pregnancy outcomes after progesterone administration. In this retrospective study, we included women who underwent a single frozen-thawed euploid blastocyst transfer using a natural cycle with luteal support and demonstrated that the clinical pregnancy rate increased significantly with increasing endometrial thickness from the progesterone administration day to the blastocyst transfer day. When the endometrial thickness change ratio was divided into three groups with a cutoff value of 0, the clinical pregnancy rate of the women with unchanged or expanded endometrial thickness was significantly higher than that of women with compacted endometrial thickness. However, with the increase in the endometrial thickness change ratio, no significant difference in the miscarriage rate or live birth rate was found (*P* > .05). In addition, the data also showed that the endometrium of patients with sharply decreased endometrial thickness in the luteal phase was good initially; however, the final result was not optimistic, suggesting that monitoring endometrial thickness after progesterone administration is very important.

To our knowledge, few studies have evaluated the influence of endometrial thickness changes after progesterone administration on the outcome of FET, and the conclusions are not consistent. Two cohort studies by Haas et al. [21] and Zilberberg et al. [20] from Canada included women who used hormone replacement therapy for endometrial preparation. As a follow-up study at the same research center, the study of Zilberberg et al. [20] used euploid blastocysts for FET. The results showed that endometrial compaction was associated with higher ongoing pregnancy rates, and with increasing percent compaction, ongoing pregnancy rates increased significantly. This was different from the conclusion of this study that the clinical pregnancy rate of women with unchanged or expanded endometrial thickness was higher. Notably, in addition to the endometrial preparation protocol used in their study being different from ours, the compaction rates in these two cohorts were much higher than the 25.1% (55/219) in our study. The difference in compaction rate may be because both cohort studies used transvaginal ultrasound to evaluate the endometrium on the progesterone administration day and transabdominal ultrasound to assess the endometrium prior to transfer, which is different from both being evaluated by transvaginal ultrasound, as in our study. Transvaginal ultrasound is generally believed to be more accurate than transabdominal ultrasound in measuring endometrial thickness, which may also explain the contrasting results of the studies.

Subsequently, Bu et al. [23] and Ye et al. [24] evaluated the relationship between the endometrial thickness change ratio and clinical pregnancy rate and live birth rate in natural cycles and hormone replacement therapy cycles, respectively. Among them, the study of Bu et al. [23] used high-quality blastocysts for transfer, concluding that the increase in endometrial thickness after progesterone administration was related to better pregnancy outcomes. As a follow-up study at the same center that only included PGT cycles, our research results were basically consistent with those of Bu et al. [23]. In the study of Ye et al. [24], embryos on the third day were transferred. The results showed that regardless of how endometrial thickness changed after progesterone administration, it had no significant effect on the clinical pregnancy rate and live birth rate in the FET cycles. Although this study also holds that the endometrial thickness change rate was not related to the live birth rate, it had a positive effect on the clinical pregnancy rate, which may be because we used euploid blastocysts on the fifth or sixth day after a biopsy unlike their use of embryos for transfer. More recently, another cohort study by Riestenberg et al. [22], including 259 single euploid blastocyst transfers using hormone replacement therapy, conducted the same analysis and showed that the endometrial thickness change rate was not associated with the live birth rate or spontaneous abortion rate, which is consistent with the secondary results of this study. Interestingly, in addition to different endometrial preparation methods, the timing of endometrial evaluation was also different in these two studies. Riestenberg et al. [22] performed the second endometrial assessment on the day prior to FET, while our study evaluated it on the day of FET. However, the duration of progesterone exposure was similar when evaluating the endometrium, so we believed that this would not affect the final results. In addition, we found a significant positive correlation between the endometrial thickness change rate and the clinical pregnancy rate.

This study is contrary to two published results reporting that endometrial compaction is beneficial to the outcome in cycles of hormone replacement therapy [20, 21]. By including the natural cycles of endometrial preparation, which only involved a single euploid blastocyst transfer cycle, we demonstrated the importance of endometrial noncompaction for endometrial receptivity. To this end, we can speculate that some possible effective interventions can be used to manage the cycles of endometrial compaction, such as increasing the dose or duration of estrogen and altering the proportion of estrogen-progesterone in the luteal phase.

From a biological point of view, the change in endometrial thickness after progesterone administration may be an effective indicator of endometrial receptivity. In the natural menstrual cycle, endometrial development is different in the follicular phase and luteal phase. In the follicular phase, estrogen secreted by the follicles causes the endometrial thickness to increase continuously, the endometrial glands to grow and the blood vessels to grow and bend [29]. In the luteal phase, the traditional belief is that the endometrium will continue to thicken, and glands and blood vessels will continue to grow under the action of estrogen and progesterone secreted by the corpus luteum, which is consistent with our conclusion that patients with increased endometrial thickness are more likely to become pregnant. However, some studies have shown that after ovulation the endometrium stops proliferating under the influence of progesterone. The endometrium that continues to grow in the luteal phase may be due to progesterone receptor deficiency or progesterone resistance, which indicates that the environment of embryo implantation is suboptimal [21]. Considering the limited and contradictory conclusions in previous studies, further investigating this mechanism is necessary.

This study has several advantages. First and foremost, we limited the population to women who used a single euploid blastocyst transfer, which enabled us to eliminate the effect of embryo ploidy status on the outcomes and focus only on the implanted and pregnant endometrium. Second, as far as we know, under the premise of euploid blastocyst transfer, no previous study on the use of natural cycles to prepare the endometrium has studied the changes in endometrial thickness after progesterone administration. Third, we collected more variables and adjusted for more variables. At the same time, all cycles used the same ovarian stimulation regimen (MLSL) and standardized endometrial preparation regimen (natural cycle) to control the potential confounders as much as possible and to ensure the reliability of the results. Fourth, we used transvaginal ultrasound to evaluate all endometrial, and it is a more accurate method for measuring endometrial thickness than transabdominal ultrasound. Fifth, our study uses actual clinical data, and the retrospective nature of the study can avoid observation bias. Finally, with the widespread use of FET and PGT, choosing to study the relationship between the endometrium and outcomes has a wide range of clinical applicability.

This study also had some limitations. First, as this was a retrospective study, we could not collect and control for all confounding factors. However, we used multiple regression analysis to adjust and control for confounders between groups, and several adjustment models were presented in the results, which supports the robustness of our results. Second, each physician in our center is experienced and can perform ultrasound examinations under standard operating procedures. Although the endometrium of each patient was evaluated by the same doctor during the study period, the differences in the measurement of endometrial thickness between observers may result in some bias in this study. However, any potential measurement biases will be equally distributed among all participants, so we do not think that this will affect our main results. Third, in the current study, cycles with a high degree of compaction had unexpectedly lower clinical pregnancy rates, and a relationship between endometrial compactness and the miscarriage rate or live birth rate was not found. However, considering that the cycles of compaction in our cohort constituted only 25.1% (55/219), this result should be interpreted carefully. Fourth, in this study, we only included women who received a natural cycle to prepare the endometrium for a single frozen-thawed euploid blastocyst transfer. For the general infertility population, a large part of infertility is caused by aneuploidy of embryos [4]. As the embryos of these patients have not been biopsied, the impact of embryonic factors on pregnancy outcomes could not be ruled out. Therefore, extending the application to the general infertile population will affect the rigor of the conclusion. In the future, further studying the changes in the endometrium in different types of transfer cycles is still necessary. Finally, the sample size of this paper is limited, and we will accumulate more samples for further research in the future.

In conclusion, we studied the relationship between the endometrial thickness change ratio and the clinical outcomes after progesterone administration. The results showed that the endometrial thickness of most patients expanded or remained unchanged on the transfer day compared with on the progesterone administration day in the cycles using single euploid blastocyst transfer and preparing the endometrium with a natural cycle. Compared with the cycles of endometrial compaction, endometrial noncompaction (including non-change and expansion) resulted in a significant increase in the clinical pregnancy rate. The significant difference in the clinical pregnancy rate between compacted and uncompacted endometrium indicated that monitoring endometrial thickness after progesterone administration and before transfer is necessary. This study only included euploid blastocyst transfer in the natural cycle with luteal support, which may be one of the reasons why our results are different from those of previous studies. Additional multicenter prospective randomized clinical trials are needed to confirm our results to determine whether the transfer would be cancelled if endometrial compaction occurs in the natural cycle.

## Supplementary Information

**Additional file 1 **: **Supplementary Table 1.** Indications for PGT-SR and PGT-M.

## Data Availability

All data supporting the conclusion of this article are included.

## Abbreviations

PGT: Preimplantation genetic testing
WOI: Window of implantation
hCG: Human chorionic gonadotrophin
FET: Frozen-thawed embryo transfer
PGD: Preimplantation genetic diagnosis
ICSI: Intracytoplasmic sperm injection
BMI: Body mass index
AMH: Anti-Müllerian hormone

## References

[1] Mahajan N (2015). Endometrial receptivity array: clinical application. J Hum Reprod Sci.

[2] Kuliev A, Rechitsky S (2017). Preimplantation genetic testing: current challenges and future prospects. Expert Rev Mol Diagn.

[3] Bashiri A, Halper KI, Orvieto R (2018). Recurrent implantation failure-update overview on etiology, diagnosis, treatment and future directions. Reprod Biol Endocrinol.

[4] von Grothusen C, Lalitkumar S, Boggavarapu NR, Gemzell-Danielsson K, Lalitkumar PG (2014). Recent advances in understanding endometrial receptivity: molecular basis and clinical applications. Am J Reprod Immunol.

[5] Yuan X, Saravelos SH, Wang Q, Xu Y, Li TC, Zhou C (2016). Endometrial thickness as a predictor of pregnancy outcomes in 10787 fresh IVF-ICSI cycles. Reprod BioMed Online.

[6] Zhang X, Chen CH, Confino E, Barnes R, Milad M, Kazer RR (2005). Increased endometrial thickness is associated with improved treatment outcome for selected patients undergoing in vitro fertilization-embryo transfer. Fertil Steril.

[7] Gallos ID, Khairy M, Chu J, Rajkhowa M, Tobias A, Campbell A (2018). Optimal endometrial thickness to maximize live births and minimize pregnancy losses: analysis of 25,767 fresh embryo transfers. Reprod BioMed Online.

[8] Zollner U, Zollner KP, Specketer MT, Blissing S, Muller T, Steck T (2003). Endometrial volume as assessed by three-dimensional ultrasound is a predictor of pregnancy outcome after in vitro fertilization and embryo transfer. Fertil Steril.

[9] Gingold JA, Lee JA, Rodriguez-Purata J, Whitehouse MC, Sandler B, Grunfeld L (2015). Endometrial pattern, but not endometrial thickness, affects implantation rates in euploid embryo transfers. Fertil Steril.

[10] Zhao J, Zhang Q, Wang Y, Li Y (2014). Endometrial pattern, thickness and growth in predicting pregnancy outcome following 3319 IVF cycle. Reprod BioMed Online.

[11] Merce LT, Barco MJ, Bau S, Troyano J (2008). Are endometrial parameters by three-dimensional ultrasound and power Doppler angiography related to in vitro fertilization/embryo transfer outcome?. Fertil Steril.

[12] Wang L, Qiao J, Li R, Zhen X, Liu Z (2010). Role of endometrial blood flow assessment with color Doppler energy in predicting pregnancy outcome of IVF-ET cycles. Reprod Biol Endocrinol.

[13] Craciunas L, Gallos I, Chu J, Bourne T, Quenby S, Brosens JJ (2019). Conventional and modern markers of endometrial receptivity: a systematic review and meta-analysis. Hum Reprod Update.

[14] Laasch C, Puscheck E (2004). Cumulative embryo score, not endometrial thickness, is best for pregnancy prediction in IVF. J Assist Reprod Genet.

[15] Barker MA, Boehnlein LM, Kovacs P, Lindheim SR (2009). Follicular and luteal phase endometrial thickness and echogenic pattern and pregnancy outcome in oocyte donation cycles. J Assist Reprod Genet.

[16] Zhang T, Li Z, Ren X, Huang B, Zhu G, Yang W (2018). Endometrial thickness as a predictor of the reproductive outcomes in fresh and frozen embryo transfer cycles: A retrospective cohort study of 1512 IVF cycles with morphologically good-quality blastocyst. Medicine..

[17] Liu KE, Hartman M, Hartman A, Luo ZC, Mahutte N (2018). The impact of a thin endometrial lining on fresh and frozen-thaw IVF outcomes: an analysis of over 40 000 embryo transfers. Hum Reprod.

[18] Kasius A, Smit JG, Torrance HL, Eijkemans MJ, Mol BW, Opmeer BC (2014). Endometrial thickness and pregnancy rates after IVF: a systematic review and meta-analysis. Hum Reprod Update.

[19] Griesinger G, Trevisan S, Cometti B (2018). Endometrial thickness on the day of embryo transfer is a poor predictor of IVF treatment outcome. Hum Reprod Open..

[20] Zilberberg E, Smith R, Nayot D, Haas J, Meriano J, Barzilay E (2020). Endometrial compaction before frozen euploid embryo transfer improves ongoing pregnancy rates. Fertil Steril.

[21] Haas J, Smith R, Zilberberg E, Nayot D, Meriano J, Barzilay E (2019). Endometrial compaction (decreased thickness) in response to progesterone results in optimal pregnancy outcome in frozen-thawed embryo transfers. Fertil Steril.

[22] Riestenberg C, Quinn M, Akopians A, Danzer H, Surrey M, Ghadir S (2021). Endometrial compaction does not predict live birth rate in single euploid frozen embryo transfer cycles. J Assist Reprod Genet.

[23] Bu Z, Yang X, Song L, Kang B, Sun Y (2019). The impact of endometrial thickness change after progesterone administration on pregnancy outcome in patients transferred with single frozen-thawed blastocyst. Reprod Biol Endocrinol.

[24] Ye J, Zhang J, Gao H, Zhu Y, Wang Y, Cai R (2020). Effect of endometrial thickness change in response to progesterone administration on pregnancy outcomes in Frozen-Thawed embryo transfer: analysis of 4465 cycles. Front Endocrinol..

[25] Li G, Wu Y, Niu W, Xu J, Hu L, Shi H (2020). Analysis of the number of euploid embryos in preimplantation genetic testing cycles with early-follicular phase long-acting gonadotropin-releasing hormone agonist long protocol. Front Endocrinol..

[26] Shi D, Xu J, Niu W, Liu Y, Shi H, Yao G (2020). Live births following preimplantation genetic testing for dynamic mutation diseases by karyomapping: a report of three cases. J Assist Reprod Genet.

[27] Zegers-Hochschild F, Adamson GD, Dyer S, Racowsky C, de Mouzon J, Sokol R (2017). The international glossary on infertility and fertility care, 2017. Fertil Steril.

[28] Farrar D, Fairley L, Santorelli G, Tuffnell D, Sheldon TA, Wright J (2015). Association between hyperglycaemia and adverse perinatal outcomes in south Asian and white British women: analysis of data from the born in Bradford cohort. Lancet Diabetes Endocrinol.

[29] Alcazar JL (2006). Three-dimensional ultrasound assessment of endometrial receptivity: a review. Reprod Biol Endocrinol.

