# Supplementary material for: Effect of endometrial thickness changes on clinical pregnancy rates after progesterone administration in a single frozen-thawed euploid blastocyst transfer cycle using natural cycles with luteal support for PGT-SR- and PGT-M-assisted reproduction: a retrospective cohort study
Source: Reprod Biol Endocrinol. 2021 Oct 9;19:154. doi: 10.1186/s12958-021-00841-x (PMC8501735; doi:10.1186/s12958-021-00841-x)
Supplement: Supplementary file 1 — Additional file 1 : Supplementary Table 1. Indications for PGT-SR and PGT-M. [file 12958_2021_841_MOESM1_ESM.docx]

**Supplementary Table 1** Indications for PGT-SR and PGT-M.

| PGD: Preimplantation genetic diagnosis uses the same process to detect a specific disease that is likely to be passed on from parents to their offspring. Preimplantation genetic diagnosis involves the genetic detection of specific gene mutations in embryos when one or both biological parents have realized the possible existence of genetic abnormalities. | | |
| --- | --- | --- |
| Classification of PGD | PGT-SR | PGT-M |
| Indications | Chromosome structural abnormality | Single gene hereditary disease |
| Included in this article | Chromosomal rearrangement  (including Reciprocal translocation and Robertsonian translocation). | CAH, Alport syndrome, DMD, Joubert syndrome, Kallmann Syndrome, albinism, PKU, PA, FXS, thalassaemia, [polycystic kidney](https://www.hujiang.com/ciku/polycystic_kidney/), HLD, HPA, PJS, SCAs, TSC, Marfan syndrome, CMD, hemophilia, SMA. |

*Note:* PGT = preimplantation genetic testing; PGT-SR = PGT for chromosomal structural rearrangements; PGT-M = PGT for monogenic/single gene defect; PGD = preimplantation genetic diagnosis; CAH = congenital adrenal cortical hyperplasia; DMD = Duchenne Muscular Dystrophy; PKU = phenylketonuria; PA = propionic acidemia; FXS = fragile X syndrome; HLD = hepatolenticular degeneration; HPA = hyperphenylalaninemia; PJS = Peutz-Jeghers syndrome; SCAs = spinocercbellar ataxias; TSC = tuberous sclerosis; CMD = congenital muscular dystrophy; SMA = Spinal muscular atrophy.
